# Supplementary material for: Transcriptional adaptation after deletion of Cdc42 in primary T cells
Source: J Cell Sci. 2025 Aug 4;138(15):jcs263826. doi: 10.1242/jcs.263826 (PMC12377712; doi:10.1242/jcs.263826)
Supplement: Supplementary information [file joces-138-263826-s1.pdf]

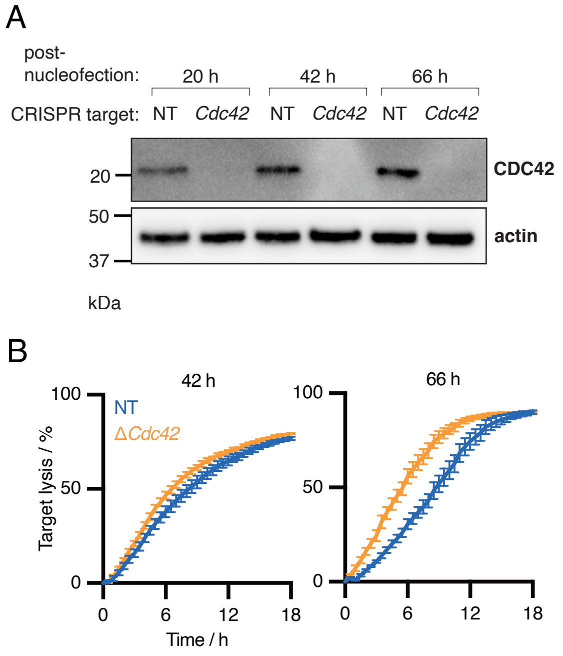

Fig. S1. Variable time for transcriptional adaption in Δ*Cdc42* CTLs. Replicate time course of daily killing assays after nucleofection with CRISPR/Cas9 RNPs, as in fig. 3A-B. No killing is observed at 20 h as CTLs recover from nucleofection. Knockout CTLs exhibit adaptation at 42 hours, the first time point after nucleofection that is testable. **A:** Immunoblot depicting loss of CDC42 in Δ*Cdc42* CTLs. **B:** IncuCyte killing assays at time points relating to A.

Journal of Cell Science • Supplementary information

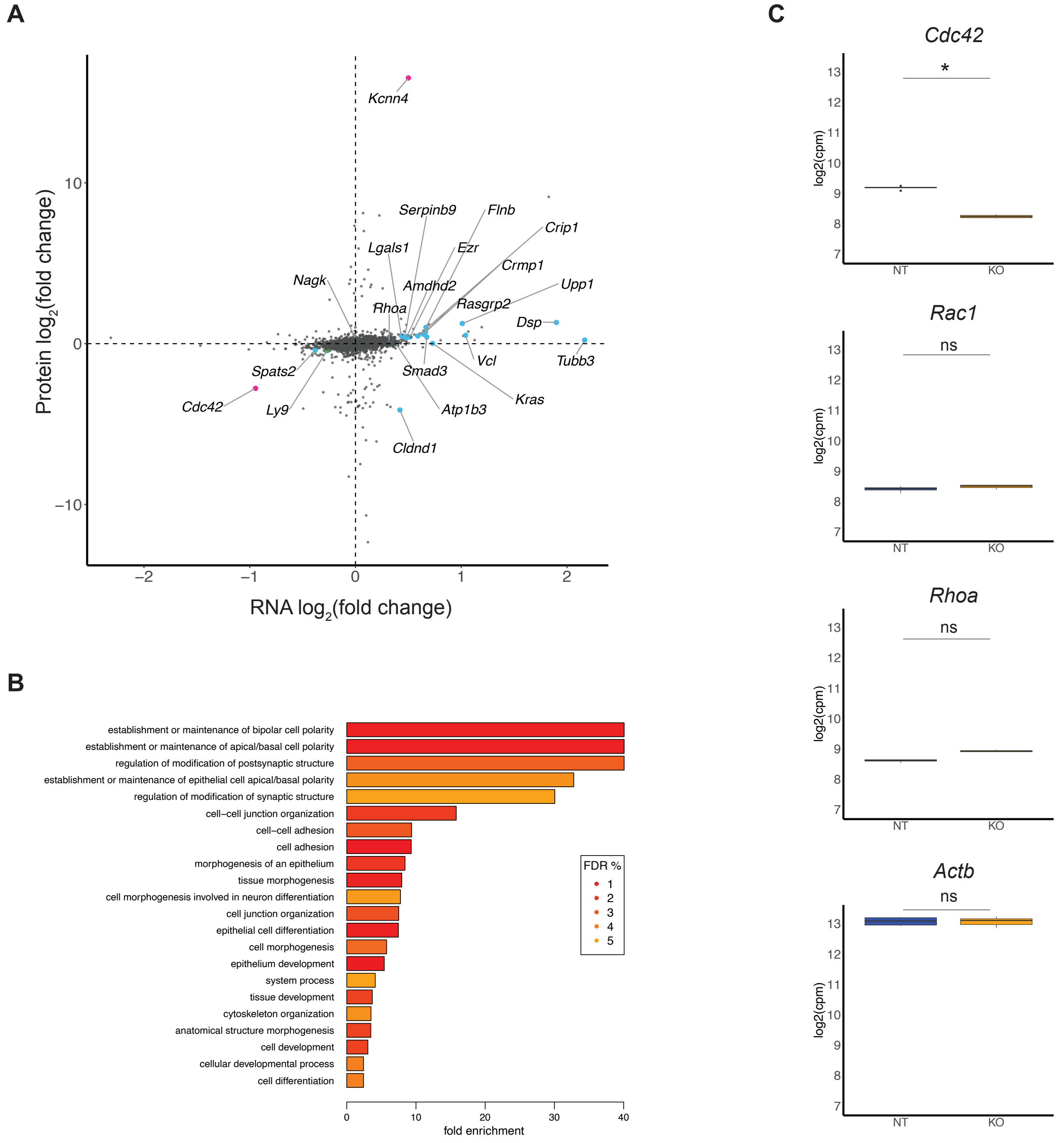

Fig. S2. Correlation between transcriptomics and proteomics after CRISPR/Cas9-mediated deletion of *Cdc42*. **A**: Effect size plot depict the $\log_2$ fold-change in gene expression in Δ*Cdc42* versus NT CTLs on the x-axis, against the $\log_2$ fold-change in protein expression in Δ*Cdc42* versus NT CTLs on the y-axis. Significantly expressed genes are coloured in blue (FDR < 0.10), significantly expressed proteins are coloured in green (FDR < 0.10), and those that are signficantly expressed in both gene and protein expression are labelled pink (FDR <0.10). All significantly expressed genes and proteins are labelled (FDR < 0.10). **B**: Gene ontology analysis of significantly expressed genes (FDR < 0.10) from (A) compared against the common 5465 genes found within both datasets. Fisher's exact test with FDR of 5% was performed in PANTHER. Barplot depicts significantly expressed GO Biological Process terms plotted against fold enrichment, coloured by FDR (%). **C**: Gene expression profiles of *Cdc42, Rac1, Rhoa* and *Actb* are depicted for Δ*Cdc42* (coloured orange) versus NT CTLs (coloured blue). Boxplot depict the median and interquatile range, dots and whiskers extend to the range of the data, *denote significant differential gene expression (FDR < 0.05).

Journal of Cell Science • Supplementary information

Table S1. Differentially expressed genes and proteins in Δ*Cdc42* CTLs. Upregulated genes/proteins are in red, downregulated in blue. If the change is not significant, the colour is dulled.

	Transcriptomics samples		Proteomics samples				
Gene symbol	***RNA log2(fold change)***	***FDR***	***Protein log2(fold change)***	***FDR***	***Protein name***	***Protein function***	***Potential compensatory function?***
Cdc42	-0.9448	0.0006	-2.7802	0.0989	Cell division control protein 42	Rho family GTPase that activates the Arp2/3 nucleation promoting factor WASP	n/a
Otof	3.3282	0.0088	Not in dataset		Otoferlin	Key calcium ion sensor that interacts with SNARE proteins to trigger exocytosis. Possible role in recycling of endosomes.	Yes; general T cell function
Upp1	1.0098	0.0088	1.2504	0.3950	Uridine phosphorylase 1	Catabolizes uridine to generate ribose and uracil for energy or nucleotide synthesis	Yes; general T cell function
Arf2	0.8773	0.0104	Not in dataset		ADP-ribosylation factor 2	Arf-family GTPase that cooperates with Rac1 to activate Arp2/3 via WAVE	Yes; GTPase-adjacent
Sell	1.6586	0.0268	Not in dataset		L-selectin	Cell adhesion molecule and activation marker. Important for migration and T cell homing.	Yes; general T cell function
Kras	0.7296	0.0280	0.0136	1.0000	Kirsten rat sarcoma virus protein (K-ras)	Kras GTPase involved in several signal transduction pathways. Also a positive regulator of Rac protein signal transduction.	Yes; GTPase-adjacent
Drc1	1.2597	0.0280	Not in dataset		Dynein regulatory complex subunit 1	Involved in motor protein attachment to microtubules	Yes; general T cell function
Smad3	0.6740	0.0355	0.4206	0.8160	Sma- and Mad-related protein 3	Transcription factor downstream of TGFβ signalling typically associated with Treg differentiation	Yes; general T cell function
Crip1	0.5896	0.0355	0.4777	0.3938	Cystein-rich protein 1	Actin-binding protein involved in cytoskeletal structures	Yes; general T cell function
Vcl	1.0408	0.0355	0.5205	0.4522	Vinculin	Actin filament binding protein that is involved in adhesion and migration.	Yes; general T cell function
Rasgrp2	0.6398	0.0355	0.5851	0.1244	Ras guanyl-releasing protein 2	Acts as a GEF for Rap1 and Ras GTPases. Important for integrin-mediated adhesion and migration in T cells	Yes; GTPase-adjacent
Cldnd1	0.4201	0.0381	-4.1255	0.9176	Claudin domain–containing protein 1	Roles in ion homeostasis and cell-cell adhesion	Yes; general T cell function
E2f2	0.7866	0.0389	Not in dataset		Transcription factor E2F2	Transcription factor involved in cell cycle progression (G1 -> S)	Yes; general T cell function
Atp1b3	0.3835	0.0402	0.0036	1.0000	Sodium/potassium-transporting ATPase subunit beta-3	Homologs have been shown to be involved in cell adhesion and epithelial cell polarity	Yes; general T cell function
Ezr	0.5162	0.0402	0.4137	0.4855	Ezrin	Links cytoskeleton to plasma membrane, plays a role in migration and immune synapse formation	Yes; general T cell function
Dsp	1.8995	0.0402	1.3179	0.5239	Desmoplakin	Shown to activate MAPK signaling cascade in cardiomyocytes	Yes; general T cell function
Stom	1.8363	0.0402	Not in dataset		Stomatin	Roles in ion homeostasis and lipid raft regulation	Yes; general T cell function
Crmp1	0.6621	0.0408	0.7158	0.4148	Dihydropyrimidinase-related protein 1	Involved in semaphorin signaling and cytoskeleton remodelling.	Yes; general T cell function
Ly9	-0.2669	0.4465	-0.4398	0.0473	LY9 / SLAMF3	Negative regulator of T cell activation	Yes; general T cell function
Rhoa	0.3181	0.0648	0.1661	0.9121	RhoA	Rho family GTPase involved in actin cytoskeleton regulation.	Yes; GTPase-adjacent
Rhob	1.3762	0.0542	Not in dataset		RhoB		

Journal of Cell Science • Supplementary information

Table S2. crRNA information.

Target	Sequence	Source	Catalogue no.
Non-targeting control	GATACGTCGGTACCGGACCG	Horizon Discovery	U-009500-01-20
Cdc42 #1	GCAAGTTTCTCAATAGTAGA	Horizon Discovery	CM-043087-01-0002
Cdc42 #2	AGCCCTCCTTACCTGTGTGA	Horizon Discovery	CM-043087-02-0002
Cdc42 #3	TCTGTCTGTGGATAACTTAG	Horizon Discovery	CM-043087-03-0002
Cdc42 promoter	ACTATGAACAATGATGTGCA	Horizon Discovery	crRNA-1113296
Rac1	GAGTCTTACTGTCTGCGGGT	Horizon Discovery	CM-041170-01-0002
Rhoa	TTGTTCCCAACCAGGATGAT	Horizon Discovery	CM-042634-01-0002

Table S3. qPCR assay information.

Target	Assay type	Source	Assay name
Actb	PrimeTime Mini qPCR Assay	IDT	Mm.PT.39a.22214843.g
Cdc42	PrimeTime Mini qPCR Assay	IDT	Mm.PT.58.29065826
Arf2	PrimeTime Mini qPCR Assay	IDT	Mm.PT.58.14187066
Rasgrp2	PrimeTime Mini qPCR Assay	IDT	Mm.PT.58.7302537
Kcnn4	PrimeTime Mini qPCR Assay	IDT	Mm.PT.58.33479151
Rhoa	PrimeTime Mini qPCR Assay	IDT	Mm.PT.58.33052169
Rac1	PrimeTime Mini qPCR Assay	IDT	Mm.PT.58.6489586

Journal of Cell Science • Supplementary information

Table S4. Table of reagents

DRUGS	Name	Target	Effect	Working concentration	Source	Catalogue number
	CASIN	CDC42	Inhibition	5 μM	Merck	SML1253
	NSC23766	Rac1	Inhibition	100 μM	Tocris	2161
**DYES**	**Name**	**Target**	**Use**	**Working concentration**	**Source**	**Catalogue number**
	CellTracker™ Green CMFDA Dye	Cytoplasm	Labelling for migration assay	1 μg/mL	ThermoFisher	C2925
	LIVE/DEAD™ Fixable Yellow Dead Cell Stain	Dead cells	Flow	1:400 (flow)	ThermoFisher	L34959
**ANTIBODIES**	**Target**	**Host species**	**Clone**	**Working concentration**	**Source**	**Catalogue number**
	CD3ε (mouse), activating	Syrian hamster	eBio500A2	1 μg/mL	ThermoFisher	14-0033-82
	CD8a (mouse)	Rat	53-6.7	1:400 (IF/ICC)	BioLegend	100701
	CDC42	Rabbit	Polyclonal	1:2000 (WB)	ProteinTech	10155-1-AP
	CDC42	Mouse	B-8	1:1000 (WB)	Santa Cruz	sc-8401
	LAMP1 (mouse)	Rat	1D4B	1:200 (IF/ICC)	DSHB	RRID: AB_528127
	LAMP1 (mouse), PE conjugated	Rat	eBio1D4B	1:200 (flow)	ThermoFisher	12-1071-82
	Mouse IgG, AlexaFluor Plus 488 conjugated	Goat	Highly cross-adsorbed polyclonal	1:500 (IF/ICC)	ThermoFisher	A32723
	Mouse IgG, AlexaFluor Plus 488 conjugated	Goat	Superclonal™	1:500 (IF/ICC)	ThermoFisher	A55058
	Phospho-p44/42 MAPK (Erk1/2) (Thr202/Tyr204)	Rabbit	D13.14.4E	1:2000 (WB)	Cell Signaling Technology	4370
	Rabbit IgG, AlexaFluor Plus 647 conjugated	Goat	Highly cross-adsorbed polyclonal	1:500 (IF/ICC)	ThermoFisher	A32733
	Rat IgG, AlexaFluor 546 conjugated	Goat	Cross-adsorbed polyclonal	1:500 (IF/ICC)	ThermoFisher	A11081
	Rat IgG, AlexaFluor Plus 555 conjugated	Goat	Highly cross-adsorbed polyclonal	1:500 (IF/ICC)	ThermoFisher	A48263
	β-actin	Mouse	AC-15	1:2000 (WB)	Sigma	A5441
	γ-tubulin	Mouse	GTU88	1:250 (IF/ICC)	Merck	T6557
	γ-tubulin	Rabbit	Polyclonal	1:200 (IF/ICC)	Merck	T5192

Journal of Cell Science • Supplementary information

Figure 1J

Anti-actin

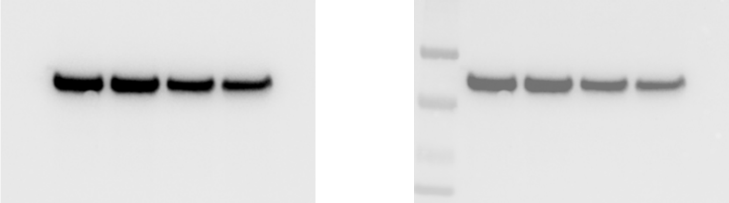

Anti-CDC42

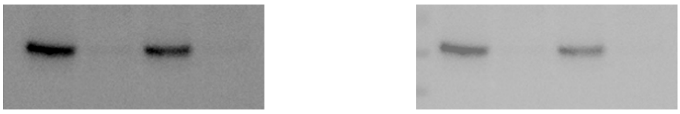

Anti-phospho-ERK1/2

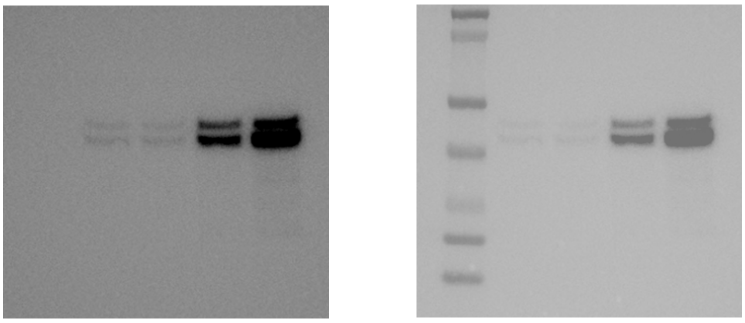

Figure 3A

Anti-alpha-tubulin

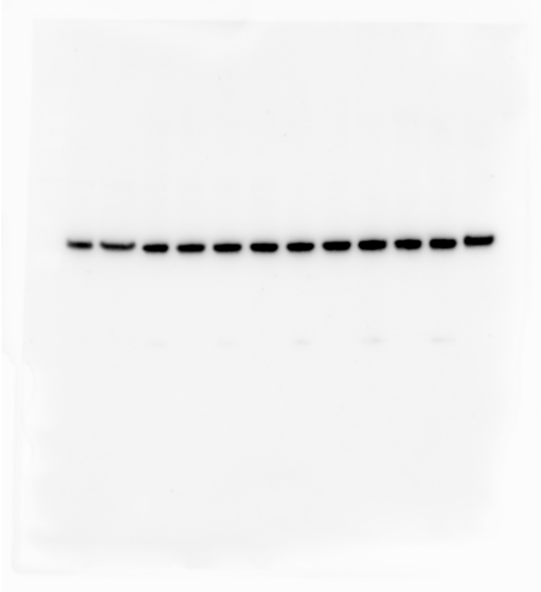

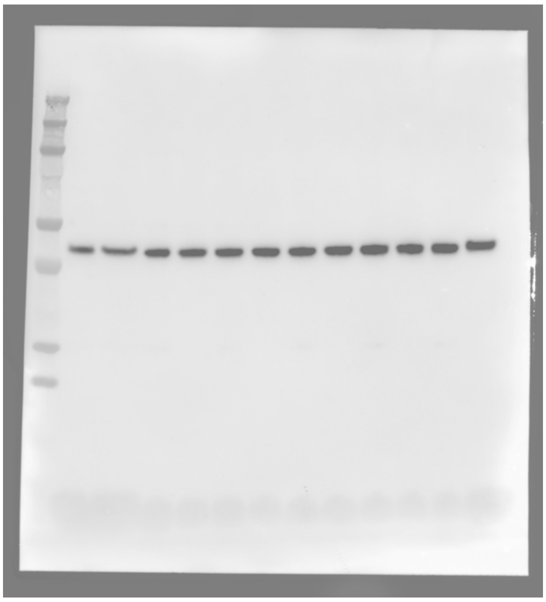

Low contrast

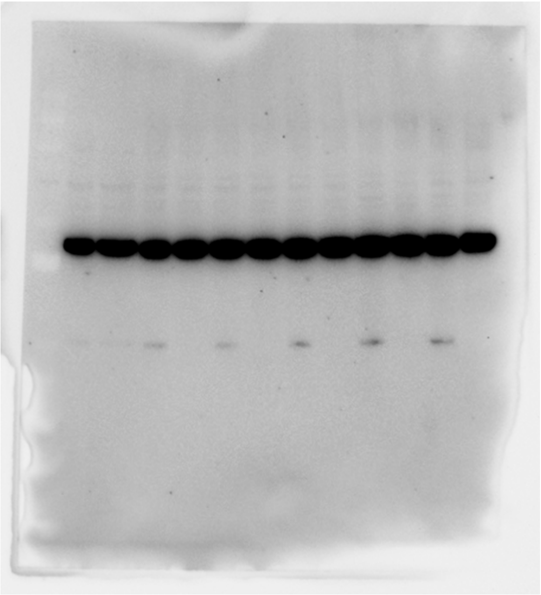

Journal of Cell Science • Supplementary information

Anti-CDC42

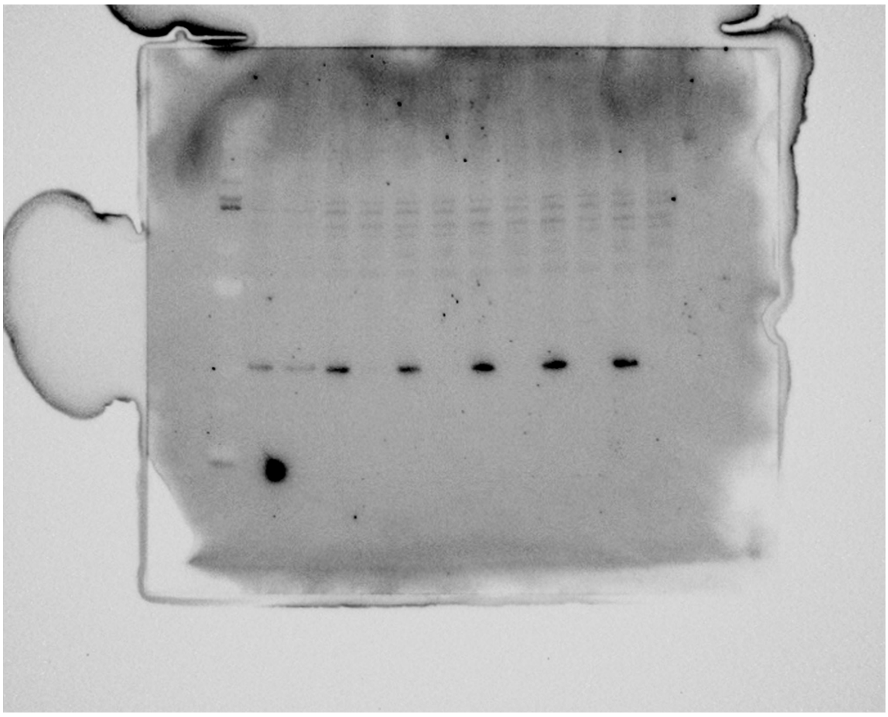

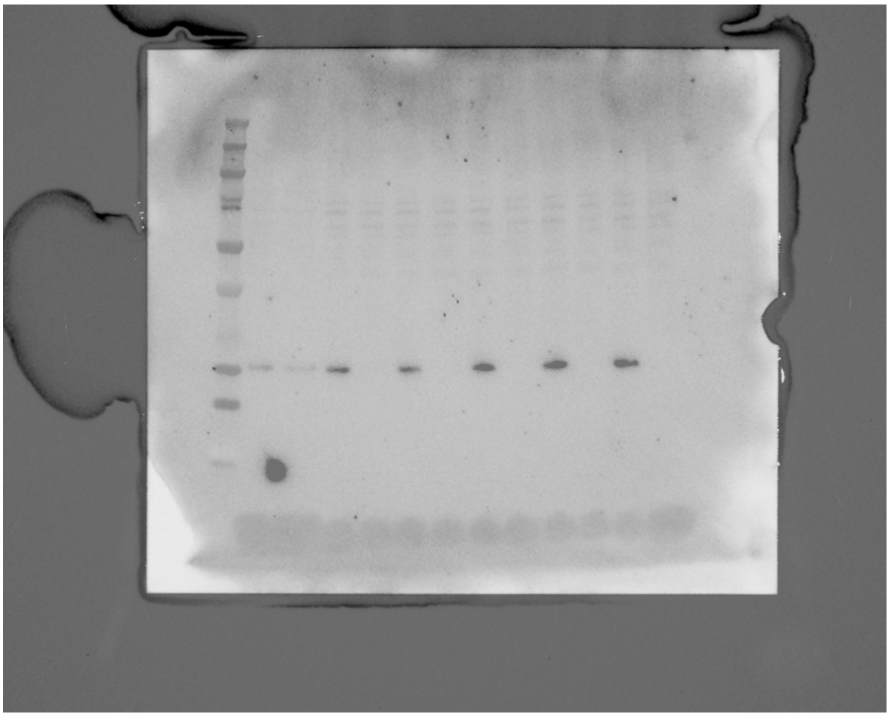

Figure S1A Anti-alpha-tubulin

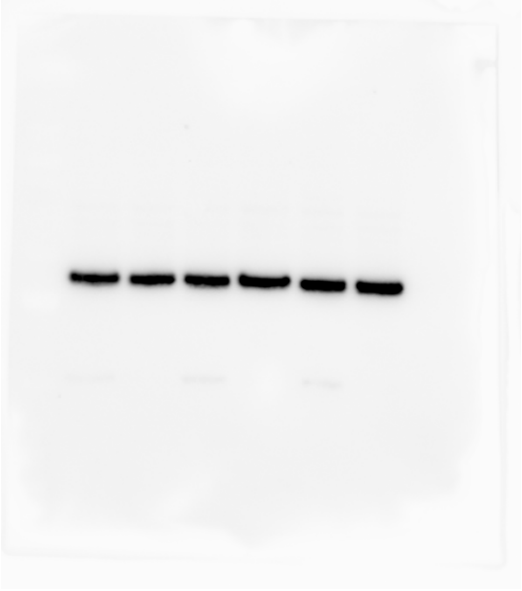

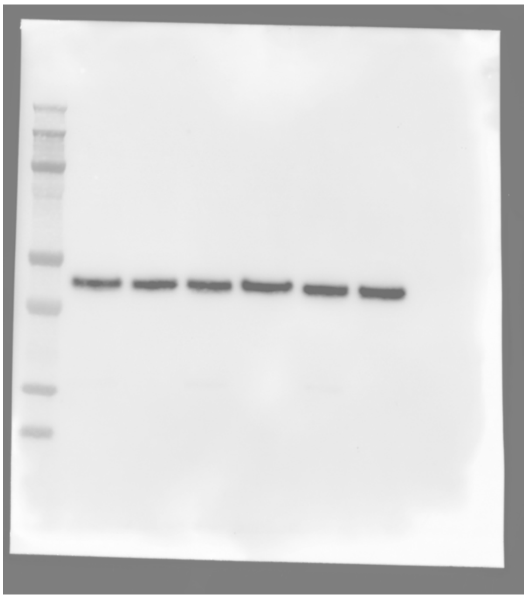

Low contrast

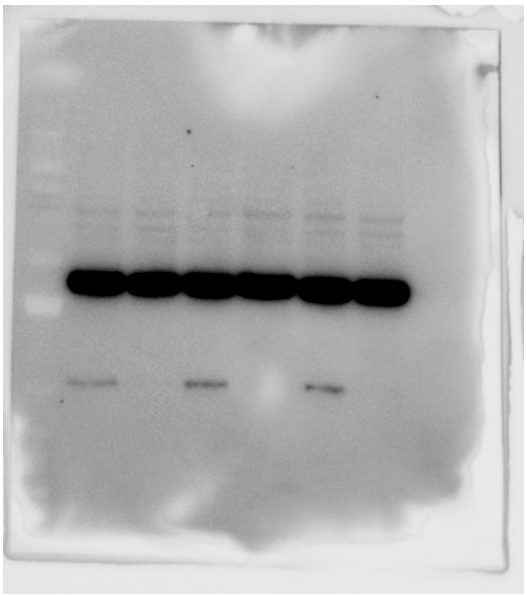

Journal of Cell Science • Supplementary information

Anti-CDC42

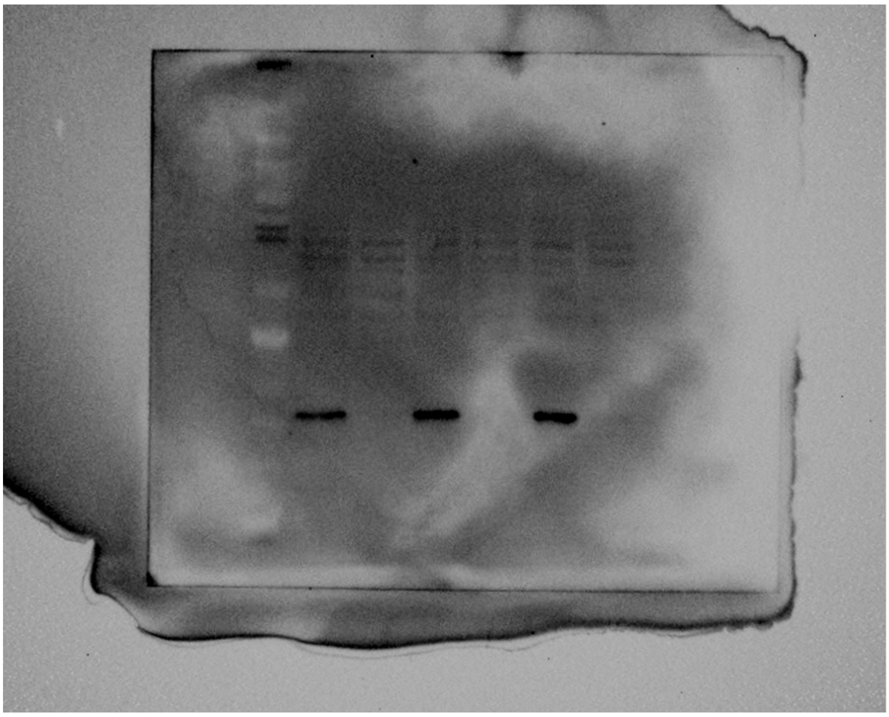

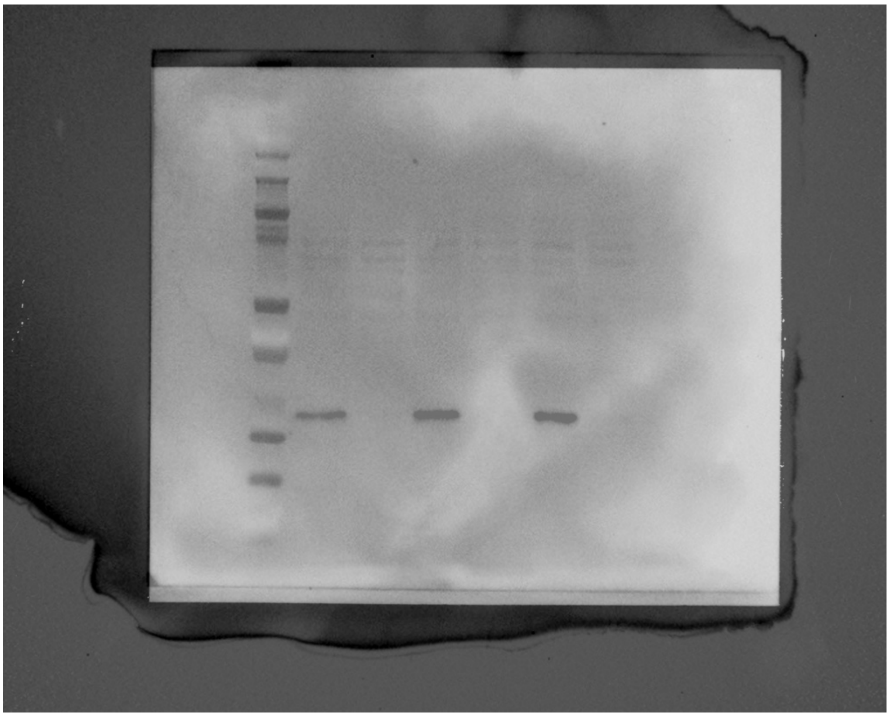

Journal of Cell Science • Supplementary information

Figure S1C

Anti-calnexin

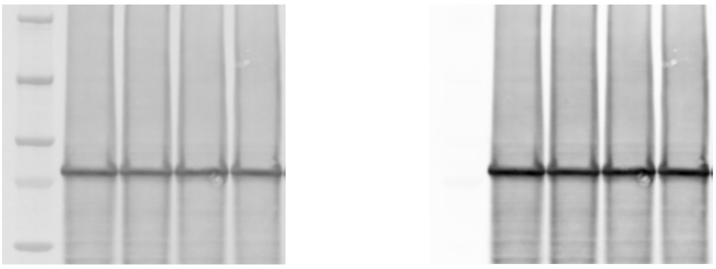

Anti-phospho-ERK1/2

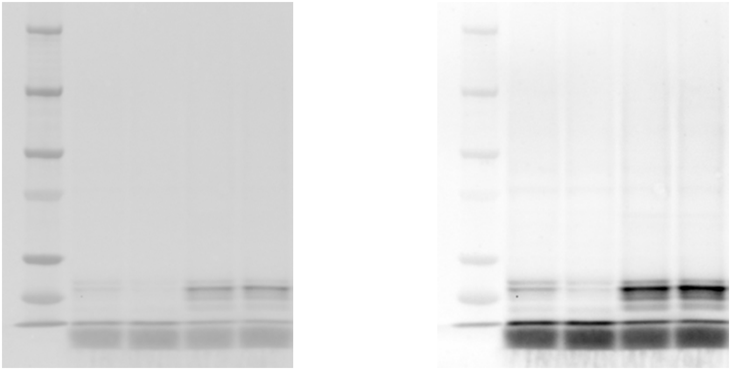

Fig. S3. Blot transparency

Chemiluminescence alone is shown on the left, composite with colorimetric channel (for molecular weight markers) is shown on the right.

Journal of Cell Science • Supplementary information
